# Two-devices-in-one-channel method using biopsy forceps for ultrasonography-guided hepaticogastrostomy reintervention

**DOI:** 10.1055/a-2599-7275

**Published:** 2025-06-13

**Authors:** Akito Furuta, Shunsuke Omoto, Hironori Tanaka, Takashi Koriyama, Mamoru Takenaka, Taro Inoue, Wataru Ono

**Affiliations:** 1 145700 Department of Gastroenterology, Kobe Tokushukai Hospital, Kobe, Japan; 2 Department of Gastroenterology, Kishiwada Tokushukai Hospital, Kishiwada, Japan; 3 Department of Gastroenterology and Hepatology, Kindai University Faculty of Medicine, Osakasayama, Japan

Reintervention following endoscopic ultrasonography-guided hepaticogastrostomy (EUS-HGS) remains challenging 1 . Several reintervention techniques have been described in the literature 2 3 4 5 . A particular technical difficulty arises when the endoscope position is unstable, especially when the HGS stent is placed in segment B2, where the proximity of the puncture site to the esophagus complicates the approach.

Herein, we report a case in which biopsy forceps were used to successfully facilitate guidewire insertion through the side hole of a type IT stent (Gadelius Medical, Tokyo, Japan) in segment B2, using the two-devices-in-one-channel technique ( Video 1 ).

The biopsy forceps facilitated guidewire insertion for ultrasonography-guided hepaticogastrostomy reintervention through the side hole of a plastic stent placed in segment B2, using the two-devices-in-one-channel method.Video 1

The patient, a 39-year-old man with alcoholic chronic pancreatitis, had undergone biliary plastic stent placement for distal bile duct stricture 8 years previously. Computed tomography revealed a stent retained in the common bile duct (CBD) with a large integrated stone ( Fig. 1 ). We successfully removed the stent via initial endoscopic retrograde cholangiopancreatography (ERCP). However, a severe stricture persisted in the distal bile duct. When balloon dilation proved inadequate, transpapillary ERCP was abandoned in favor of EUS-HGS. An IT stent was deployed in segment B2 to facilitate stone removal.

**Fig. 1:**
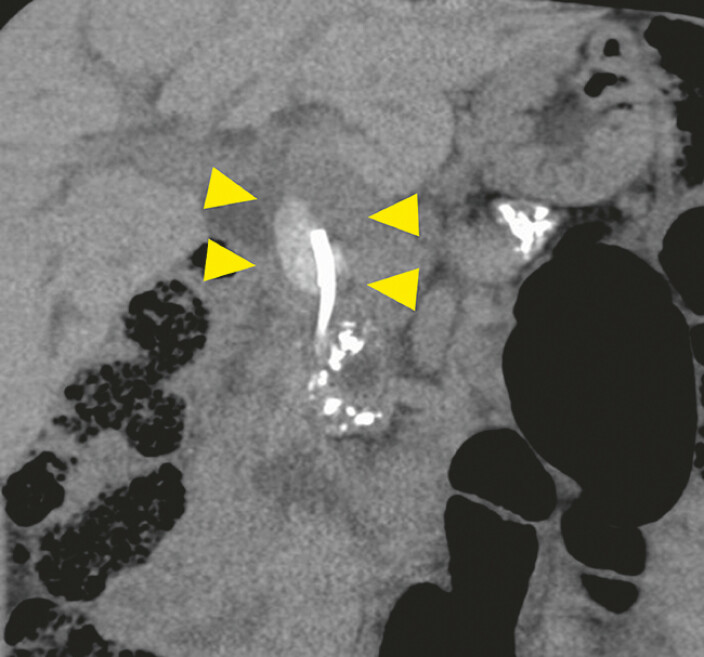
Computed tomography of a patient with chronic pancreatitis revealed a retained stent in the common bile duct with a large integrated stone (arrow).

For reintervention, we used an ERCP scope (TJF-Q290V; Olympus, Tokyo, Japan). The position of the plastic stent near the esophagogastric junction created access difficulties ( Fig. 2 ). The stent flap was resected using a snare to facilitate guidewire insertion. Although the guidewire successfully entered the stent, its passage was impeded by the kinking at the gastric wall ( Fig. 3 ). Biopsy forceps were used to grasp and retract the stent, thereby stabilizing it and correcting the kink, enabling guidewire passage ( Fig. 4 ) and successful CBD stone removal ( Fig. 5 ).

**Fig. 2:**
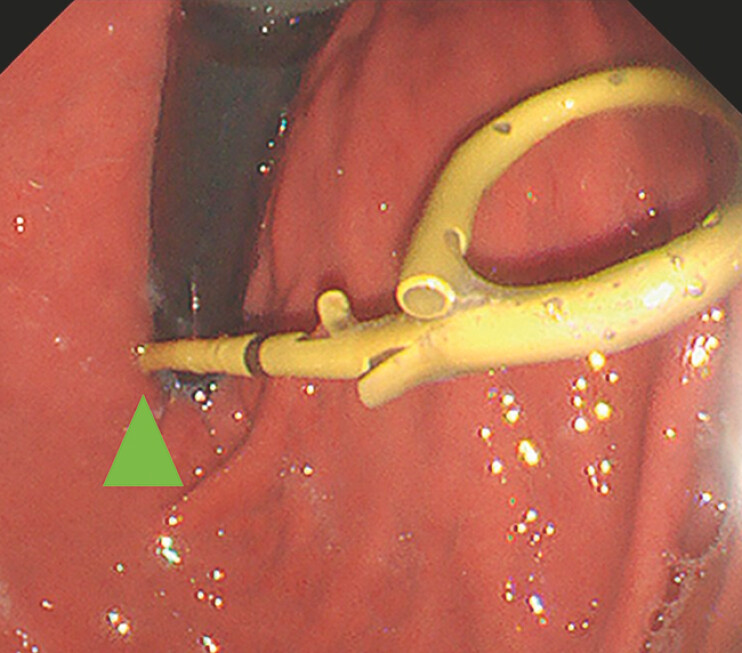
The position of the plastic stent near the esophagogastric junction (arrow) created access difficulties.

**Fig. 3:**
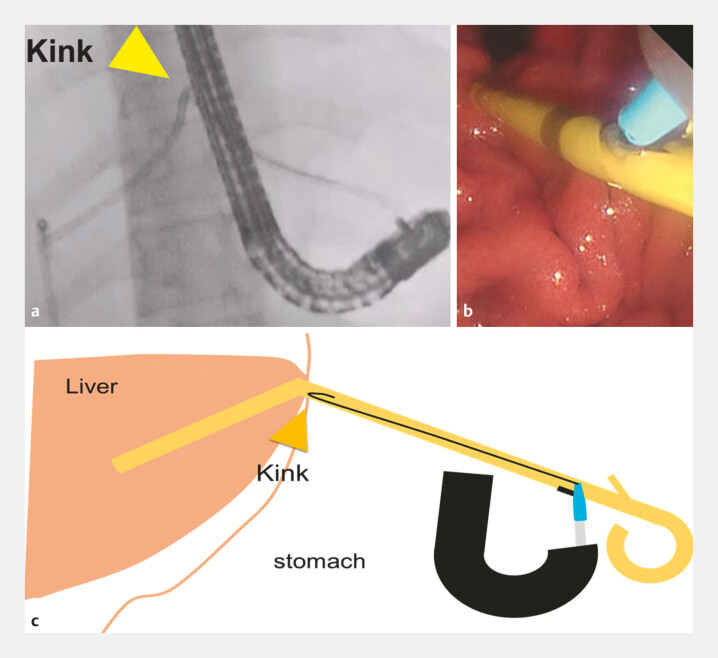
Although the guidewire successfully entered the stent, its passage was impeded by kinking at the gastric wall (arrow). **a** Fluoroscopic image. **b** Endoscopic image. **c** Schematic.

**Fig. 4:**
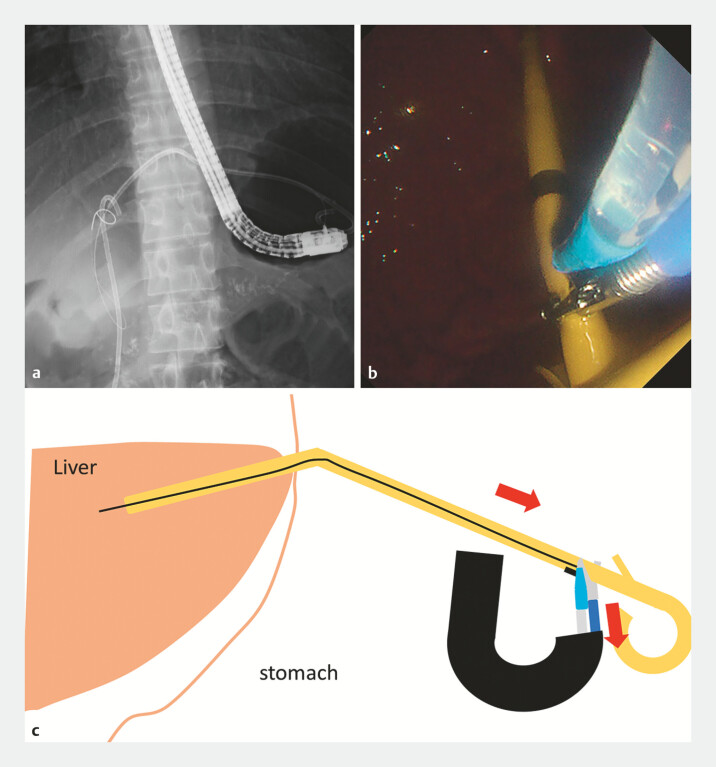
Biopsy forceps were used to grasp and retract the stent, thereby stabilizing it and correcting the kink, enabling guidewire passage. **a** Fluoroscopic image. **b** Endoscopic image. **c** Schematic.

**Fig. 5:**
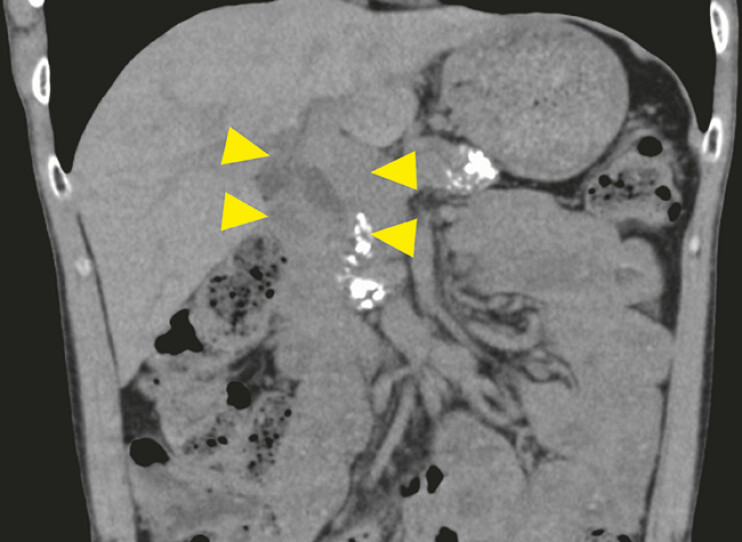
Finally, the common bile duct stone was successfully removed (arrow).

This case illustrates the utility of the two-devices-in-one-channel technique for EUS-HGS reintervention. The simultaneous use of biopsy forceps for stent manipulation while maintaining guidewire access through a single channel offers a viable solution for technically demanding cases.

Endoscopy_UCTN_Code_TTT_1AS_2AH

## Notes

Endoscopy E-Videos https://eref.thieme.de/e-videos
*E-Videos* is an open access online section of the journal *Endoscopy* , reporting on interesting cases and new techniques in gastroenterological endoscopy. All papers include a high-quality video and are published with a Creative Commons CC-BY license. Endoscopy E-Videos qualify for HINARI discounts and waivers and eligibility is automatically checked during the submission process. We grant 100% waivers to articles whose corresponding authors are based in Group A countries and 50% waivers to those who are based in Group B countries as classified by Research4Life (see: https://www.research4life.org/access/eligibility/ ). This section has its own submission website at https://mc.manuscriptcentral.com/e-videos .
